# Female Education

**Published:** 1875-02

**Authors:** 


FEMALE EDUCATION.—The matter of female education has of late been discussed with heated vigor, especially since female schools produce so many able specimens of their own. We are told that girls must be so educated that they may be able to work their way through life independently, not only as teachers, but as physicians, lawyers, civil engineers, members of Congress, Presidents, and so on. That is one extreme. The other is, that a woman, in order to remain a good woman, must not know too much. As to the second of these opinions, it seems absurd. Neither a woman nor a man can know too much. But as to the first of these extremes, something more is to be said. It is certainly true that the education of girls should enable them to work their way honorably through life. Many more occupations should be open to them and a full measure of reward should be given to them, and in this respect our public institutions should grow more and more liberal. But, on the other hand, no system of education should stimulate the desire to work their way independently and alone. We do not hesitate to declare that it is not well for woman to remain alone. It is the calling for a woman to get married, as it is in the order of things that man should feel himself destined to become a father and a husband. We should therefore condemn as equally absurd any system of education, any social order, calculated to induce young women to remain spinsters. Education should be directed to make men good husbands and good fathers, and to make women good wives and mothers. This end seems vastly more important in the case of females than in the education of men, because they are to exercise so great a range of influence upon society and upon the rising generation. It may be asked, would we have women educated alone for the drudgery of the household? We answer, by no means. We regard woman as the soul of the family home. We regard the mother as the being who holds the leading strings in her hands, and who has the highest and most exalted duties to fulfill. And for the proper fulfillment of these duties the intellect and the mind should be prepared by proper culture. Our strong-minded female friends must not take exceptions to this theory of ours. We would not lessen woman's power one iota, nor circumscribe the limits of her labor. Her "sphere" shall be as wide as she sees fit to make it, but as nature has decided that the human race shall not be extinguished, we insist upon it that she shall be fitted first of all to do her true work, of her "call" to which there can be no sort of doubt.

---

HEMORRHAGE IN AMPUTATIONS.—A new and ingenious device has been invented for preventing hemorrhage in amputations. It consists in wrapping closely around the limb elastic bands made of silk and india rubber, from below upward, to a few inches above the place where amputation or excision is to be performed. There an india rubber cord is tightly drawn around. The compression obtained by the bands empties the blood vessels on which they are brought to bear, and the circular ligature prevents the blood which has been driven back from resuming its downward course. The bands are then removed, the ligature is left in its place, and the parts on which the surgeon is to use his instruments are as bloodless as if belonging to a dead body.

---

If speaking is worth a small coin, silence is worth a large one.

